# Apoptosis Induction and Alteration of Cell Adherence in Human Lung Cancer Cells under Simulated Microgravity

**DOI:** 10.3390/ijms20143601

**Published:** 2019-07-23

**Authors:** Carlo Dietz, Manfred Infanger, Alexander Romswinkel, Florian Strube, Armin Kraus

**Affiliations:** Department of Plastic, Aesthetic and Hand Surgery, Otto-von-Guericke-University, Leipziger Strasse 44, D-39120 Magdeburg, Germany

**Keywords:** apoptosis, cell adhesion, cytoskeleton, lung neoplasms, weightlessness

## Abstract

Background: Lung cancer cells are known to change proliferation and migration under simulated microgravity. In this study, we sought to evaluate cell adherence, apoptosis, cytoskeleton arrangement, and gene expression under simulated microgravity. Methods: Human lung cancer cells were exposed to simulated microgravity in a random-positioning machine (RPM). Cell morphology and adherence were observed under phase-contrast microscopy, cytoskeleton staining was performed, apoptosis rate was determined, and changes in gene and protein expression were detected by real-time PCR with western blot confirmation. Results: Three-dimensional (3D)-spheroid formation was observed under simulated microgravity. Cell viability was not impaired. Actin filaments showed a shift in alignment from longitudinal to spherical. Apoptosis rate was significantly increased in the spheroids compared to the control. *TP53*, *CDKN2A*, *PTEN,* and *RB1* gene expression was significantly upregulated in the adherent cells under simulated microgravity with an increase in corresponding protein production for p14 and RB1. *SOX2* expression was significantly upregulated in the adherent cells, but protein was not. Gene expressions of *AKT3*, *PIK3CA,* and *NFE2L2* remained unaltered. Conclusion: Simulated microgravity induces alteration in cell adherence, increases apoptosis rate, and leads to upregulation of tumor suppressor genes in human lung cancer cells.

## 1. Introduction

Lung cancer is one of the most common malignancies of the western world. The age-standardized incidence of lung cancer ranges from 33.3 to 66.8 per 100,000 among males and 10.5 to 37.5 per 100,000 among females [1]. The disease carries a poor prognosis with a mortality rate of 46.0 per 100,000 individuals per year in the United States [2]. The advent of molecular targeted therapy has led to a paradigm shift in the treatment of lung cancer [3]. In order to develop advanced therapeutic strategies, a more detailed understanding of molecular mechanisms at play is necessary. In vitro studies may hold the key to understanding these mechanisms. 

Conventional two-dimensional (2D) cell cultures suffer from several limitations, including accelerated cell senescence [4], cytoskeletal stretching [5], and altered apoptosis rates [6]. In contrast, three-dimensional (3D)-cultured cells have shown phenotype expression that is closer to the original tissue than those grown in 2D. Compared with 2D-cultured cells, 3D-cultured hepatocytes have demonstrated enzyme expression closer to mature hepatocytes [7], while 3D-cultured mesothelioma spheroids have shown upregulation in argininosuccinate synthase 1 (ASS1) expression [8]. In addition, drug testing of 3D-cultured cells may also prove both more clinically relevant and more accurate than drug testing of 2D-cultures. Both ovarian and colorectal cancer cell spheroids have shown a higher resistance to cytostatic agents than 2D cultures [9], while lung adenocarcinoma spheroids have shown decreased radiation sensitivity [10]. These studies suggest that spheroids may prove to be a convenient and clinically relevant model for cancer drug testing. 

Simulated microgravity created by a random positioning machine (RPM) places cells in a novel state of equilibrium that allows their observation in a scaffold-free environment that is influenced by very low external forces. 

We previously demonstrated that breast and thyroid cancer cells [11,12], chondrocytes [13], and tenocytes [14] form 3D cell compounds or spheroids that are characterized by significant cytoskeletal rearrangement and changes in gene expression compared to cells grown under 1-g conditions. In certain tumors, including colorectal cancer and melanoma [15,16], simulated microgravity has been shown to increase the rate of apoptosis. Conversely, simulated microgravity decreases apoptosis in human fetal fibroblasts [17]. 

We previously showed that lung cancer cells exposed to simulated microgravity on a rotating clinostat demonstrate spheroid formation with altered cell proliferation and migration [18]. These lung cancer spheroids have the potential to serve as a valuable model to further understand cancer pathophysiology and sensitivity to various therapies. 

It is currently not known if lung cancer spheroids also exhibit cytoskeletal rearrangement, altered adherence and tumor-related gene expression, and apoptosis. In this study, we exposed human squamous lung cancer cells to simulated microgravity in a random positioning machine to investigate changes in cell adherence, cytoskeleton alteration, gene/protein expression, and apoptosis in a spheroid model. 

## 2. Results

### 2.1. Light Microscopy

**Figure 1:**
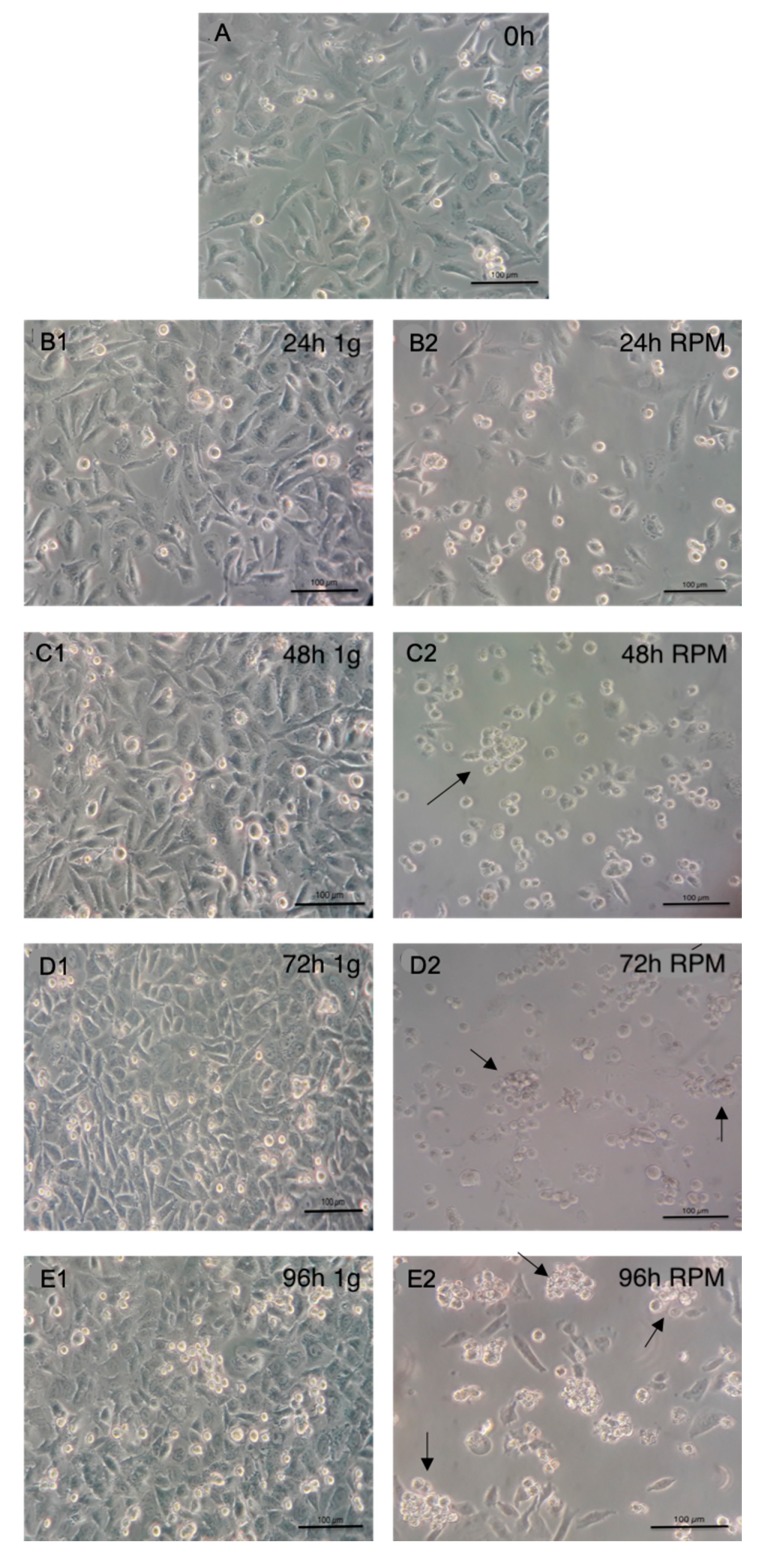
Light microscopy 100x magnification. (**A**) At starting timepoint, (**B**) at 24 h, (**C**) at 48 h, (**D**) at 72 h and (**E**) at 96 h. Control images, left; cells under simulated microgravity, right. Arrows indicate spheroids.

During the observation period, cells under simulated microgravity showed detachment from the culture surface and formation of spheroids. Cells under simulated microgravity had round to cylindrical shapes, and spheroids increased in size during ongoing exposure. In the 1-g control group, cells showed typical flat polygonal morphology and remained attached to the culture surface. Figure 1 depicts the course of spheroid formation at the beginning of the experiment (A) and after 24 h (B), 48 h (C), 72 h (D), and 96 h (E). Spheroid size (arrows) increased concomitantly with extended exposure to simulated microgravity, with cells showing no signs of impaired viability. 

### 2.2. Trypan Blue Staining

**Figure 2:**
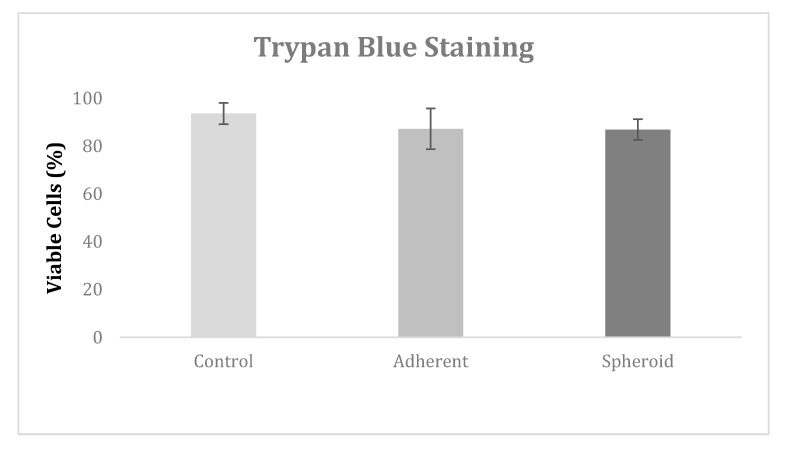
Trypan blue staining revealed no significant difference in cell viability between the 1-g control group, the adherent cells under microgravity, and the spheroids.

Trypan blue staining at 72 h revealed no significant difference in cell viability between the 1-g control group, the adherent cells under microgravity, and the spheroids. Cell viability was 93.3% in the control group, 88.0% in the adherent cells under microgravity, and 88.8% in the spheroids (Figure 2).

### 2.3. Confocal Microscopy

**Figure 3:**
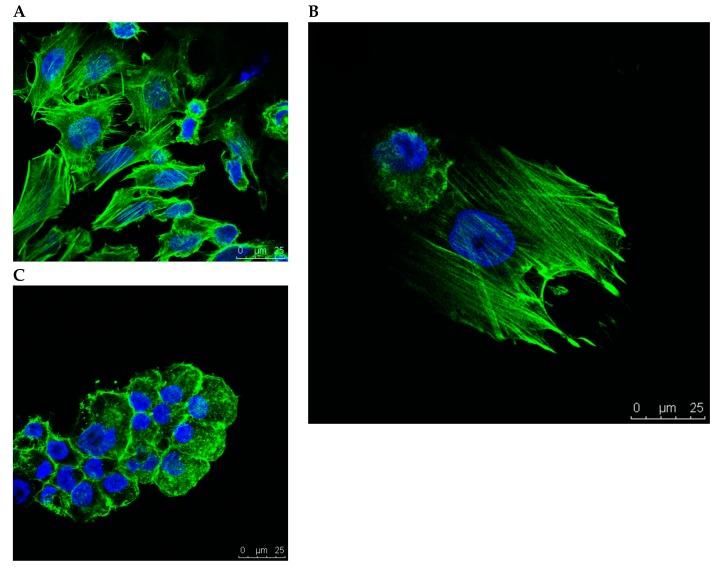
In the 1-g control group, cells showed longitudinal alignment of the actin filaments (**A**). Under simulated microgravity, cells showed spherical arrangement of the actin filaments in the outer regions of the cytoplasm, accentuated in the region of the cell membrane. This occurred in the adherent cells (**B**) and was even more pronounced in the spheroids (**C**).

In the 1-g control group, cells showed rearrangement of actin filaments towards a longitudinal alignment (Figure 3A). Under simulated microgravity, cells showed spherical arrangement of the actin filaments in the outer regions of the cytoplasm. This was accentuated in the cell membrane area of the adherent cells (Figure 3B) and even more pronounced in the spheroids (Figure 3C).

### 2.4. TUNEL-Assay

**Figure 4:**
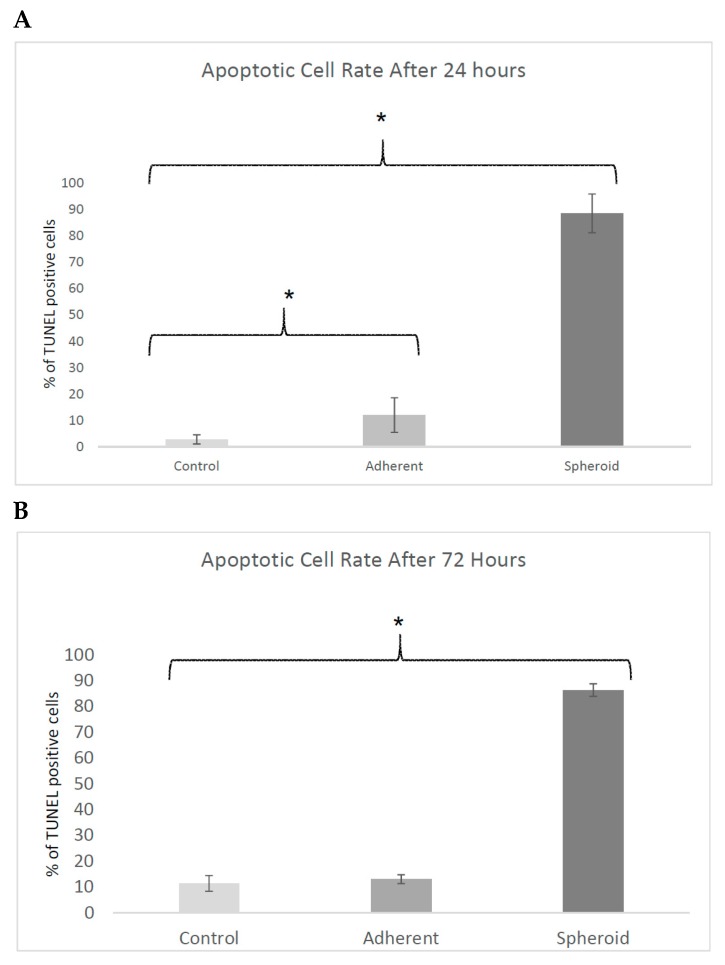
Terminal uracil-nicked end labeling (TUNEL) assays after 24 h (**A**) and after 72 h (**B**). The apoptosis rate was significantly increased in the adherent cells and in the spheroids after 24 h. After 72 h, the apoptosis rate was significantly increased in the spheroids compared to the 1-g control (*p < 0.05).

After 24 h, the apoptosis rate was 2.8% in the 1-g control, 12.0% in the adherent cells under simulated microgravity, and 88.6% in the spheroids. This was significantly different both for the adherent cells and the spheroids compared to the 1-g control (*p* < 0.05). After 72 h, the apoptosis rate was 11.4% in the 1-g control group, 13.1% in the adherent cells under simulated microgravity, and 86.4% in the spheroids. The difference was significant between spheroids and 1-g control. Values are shown in Figure 4.

### 2.5. Real Time PCR and Western Blot

**Figure 5:**
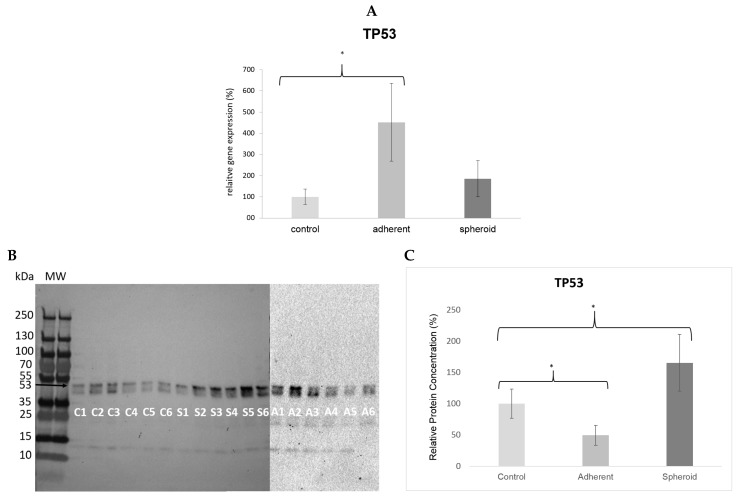
(**A**) *TP53* gene expression was significantly upregulated (4.5×, * *p* < 0.05) in the adherent cells under simulated microgravity. It was also upregulated in the spheroids (1.9×) but did not reach statistical significance. (**B**) Western blot bands showing TP53 protein production (molecular weight: 43 kD). Each lane 1–6 shows protein from one independent experiment (C: 1-g control, S: spheroids, A: adherent cells under simulated microgravity). (**C**) The bar graph shows the average density of the blots from the respective experimental group. TP53 protein production was significantly decreased in the adherent cells but significantly increased in the spheroids compared to the 1-g control (* *p* < 0.05, calculated from the average bar intensity). MW: molecular weight, kDa: kilodalton.

**Figure 6:**
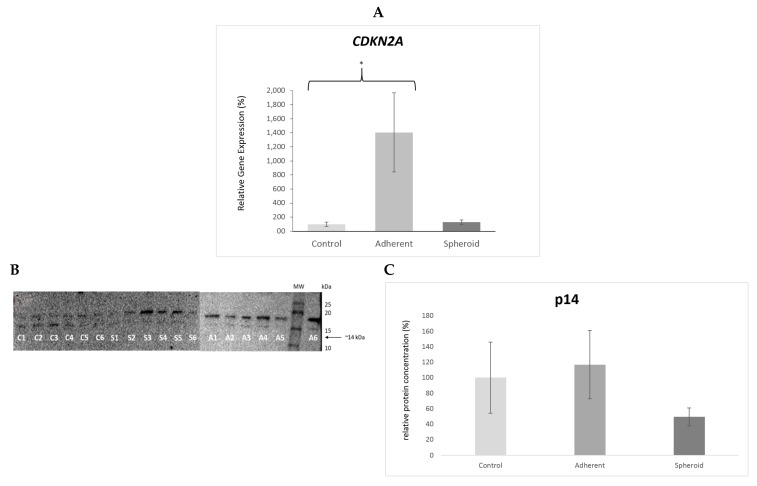
(**A**) *CDKN2A* gene expression was significantly upregulated (14.1×, * *p* < 0.05) in the adherent cells under simulated microgravity. There was no significant change in gene expression (1.3×) in the spheroids. (**B**) Western blot bands showing P14 protein production (molecular weight 14 kD). Each lane 1–6 shows protein from one independent experiment (C: 1-g control, A: adherent cells under simulated microgravity, S: spheroids). The bar graph below shows average density of the blots from the respective experimental group (controls, adherent cells under simulated microgravity, spheroids). P14 production showed slight increase in the adherent cells and slight decrease in the spheroids but without statistical significance. (**C**) Mean intensity of the bands in the respective group, intensity of controls defined as 100%.

**Figure 7:**
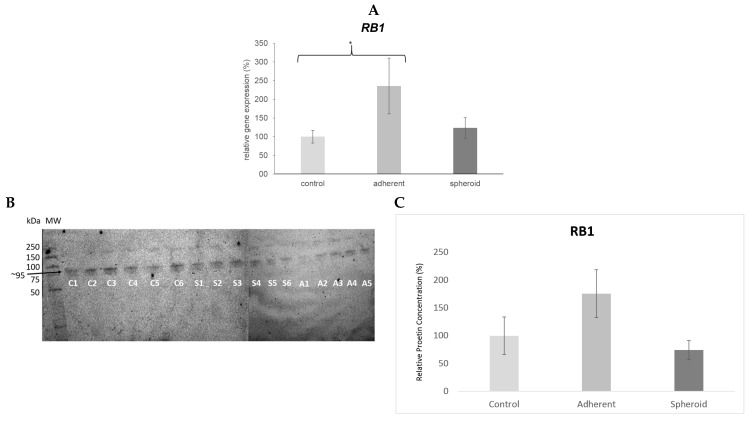
(**A**) *RB1* gene expression was significantly upregulated (2.4×, * *p* < 0.05) in the adherent cells under simulated microgravity. There was no significant change in *RB1* gene expression in the spheroids. (**B**) Western blot bands showing RB1 protein production (molecular weight 106 kD). Each lane 1–6 shows protein from one independent experiment (C: 1-g control, S: spheroids, A: adherent cells under simulated microgravity). The bar graph below shows the average density of the blots from the respective experimental group (controls, adherent cells under simulated microgravity, spheroids). RB1 protein production was increased in the adherent cells under simulated microgravity but without statistical significance. (**C**) Mean intensity of the bands in the respective group, intensity of controls defined as 100%.

**Figure 8:**
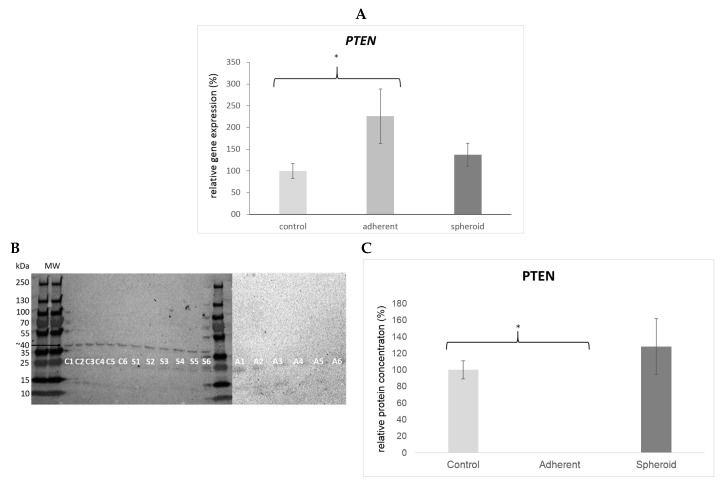
(**A**) PTEN gene expression was significantly upregulated (2.3×, * *p* < 0.05) in the adherent cells under simulated microgravity but not in the spheroids (1.4×, n.s.). (**B**) Western blot bands showing PTEN protein production (molecular weight 47 kD). Each lane 1–6 shows protein from one independent experiment (C: 1-g control, S: spheroids A: adherent cells under simulated microgravity). The bar graph below shows the average density of the blots from the respective experimental group (controls, adherent cells under simulated microgravity, spheroids). PTEN protein production was suppressed below detection level in the adherent cells under simulated microgravity and showed no significant difference compared to the 1-g-control in the spheroids. (**C**) Mean intensity of the bands in the respective group, intensity of controls defined as 100%.

**Figure 9:**
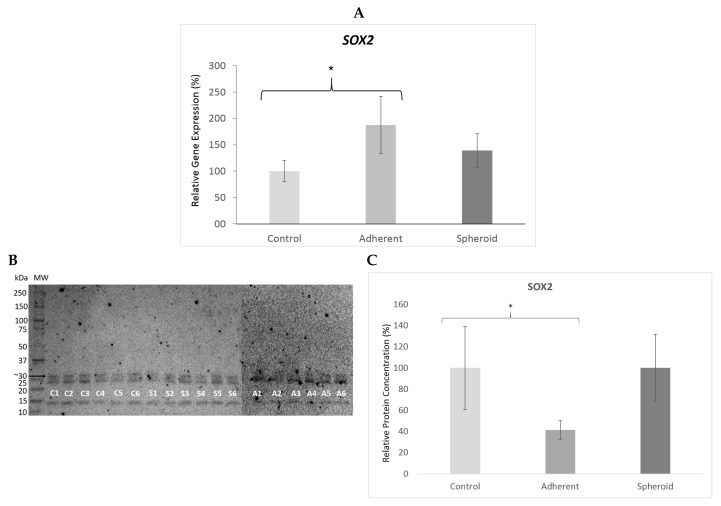
(**A**) *SOX2* gene expression was significantly upregulated in the adherent cells under simulated microgravity (1.9×, * *p* < 0.05). Upregulation did not reach statistical significance in the spheroids (1.4×, n.s.). (**B**) Western blot bands showing SOX2 protein production (molecular weight 34 kD). Each lane 1–6 shows protein from one independent experiment (C: 1-g control, S: spheroids, A: adherent cells under simulated microgravity). The bar graph below shows the average density of the blots from the respective experimental group (controls, adherent cells under simulated microgravity, spheroids). SOX2 protein production was significantly lowered in the adherent cells under simulated microgravity (*p* < 0.05) and showed no significant change in the spheroids. (**C)** Mean intensity of the bands in the respective group, intensity of controls defined as 100%.

**Figure 10:**
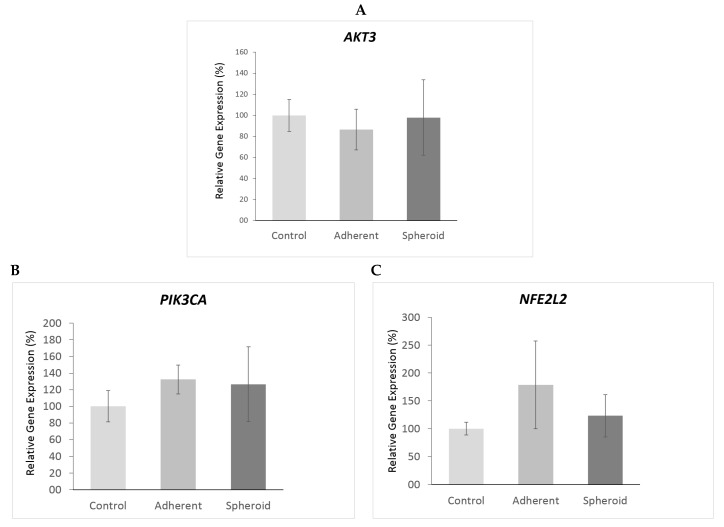
No significant changes in gene expression for *AKT3* (**A**), *PIK3CA* (**B**), or *NFE2L2* (**C**) could be observed for the adherent cells under simulated microgravity or for the spheroids. No western blot confirmation was done for the corresponding gene products.

*TP53* gene expression was significantly upregulated (4.5×, *p* < 0.05) in the adherent cells under simulated microgravity. It was also upregulated in the spheroids (1.9×) but did not reach statistical significance (Figure 5A). Western blotting revealed a significant decrease in TP53 protein content in the adherent cells (*p* < 0.05) and a significant increase in TP53 protein content in the spheroids (Figure 5B). *CDKN2A* gene expression was significantly upregulated (14.1×, *p* < 0.05) in the adherent cells under simulated microgravity (Figure 6A), while p14 protein content in the adherent cells was slightly increased (Figure 6B). There was no significant change in *CDKN2A* gene expression (1.3×) and p14 protein content in the spheroids (Figure 6B). *RB1* gene expression was significantly upregulated (2.4×, *p* < 0.05) in the adherent cells under simulated microgravity. In contrast, there was no significant change in *RB1* gene expression in the spheroids (1.2×) (Figure 7A). Rb1 protein content was increased in the adherent cells but was not statistically significant, while it was slightly decreased in the spheroids (Figure 7B). *PTEN* gene expression was significantly upregulated (2.3×, *p* < 0.05) in the adherent cells under simulated microgravity but not in the spheroids (1.4×, n.s.) (Figure 8A). PTEN protein content, in contrast, was below detection level in the adherent cells and underwent no significant change in the spheroids (Figure 8B). *SOX2* gene expression was significantly upregulated in the adherent cells under simulated microgravity (1.9×, *p* < 0.05), while *SOX2* upregulation did not reach statistical significance in the spheroids (1.4×, n.s.) (Figure 9A). SOX2 protein expression was significantly lower in the adherent cells under simulated microgravity than in the 1-g control (*p* < 0.05). SOX2 protein content in the spheroids was equal to that in the 1-g control group (Figure 9B). There were no significant changes in gene expression for *AKT3*, *PIK3CA*, and *NFE2L2* for the adherent cells under simulated microgravity or for the spheroids (Figure 10A–C). Western blotting was not performed for the corresponding proteins due to this reason. 

## 3. Discussion

Lung cancer is one of the leading causes of mortality, and there is a pressing need to improve our therapeutic armamentarium. Many believe that, unlike 3D cultures, 2D cultures cannot sufficiently simulate in vivo conditions [19]. By applying simulated microgravity to human lung cancer cells in a random positioning machine, we subjected human squamous lung cancer cells to a new 3D equilibrium state. This approach is not novel, with other authors demonstrating successful exposure of human lung cancer cells to simulated microgravity [18,20,21]. What is unique, however, is the investigation of cell adherence, cytoskeletal arrangement, and apoptosis in these squamous lung cancer spheroids. To our knowledge, this is the first study exploring these parameters. As described further below, cell adherence and apoptosis are two particular features that may have a significant relevance with regards to designing new targeted therapies. 

From our study, we observed changes in cell morphology, adherence, cytoskeletal arrangement, gene expression, protein production, and apoptosis. Together, these findings suggest that squamous lung cancer cells, not unlike connective tissue cells, may be exquisitely sensitive to external forces. 

Connective tissue cells such as tenocytes demonstrate alterations in proliferation, collagen production, and gene expression after exposure to shear [22] and a reduction in in vitro senescence and phenotype loss following the application of simulated microgravity [14]. Osteocytes have been found to possess cellular processes that are sensitive to pico Newton magnitude forces and respond with changes in intracellular Ca^2+^ signaling [23]. Airway smooth muscle cells have also been found to express a contractile phenotype in the presence of mechanical load [24]. 

We observed changes in the visual morphology of lung cancer cells in the form of detachment from the culture surface and the formation of round to elliptically shaped spheroids. Spheroid size increased with extended exposure to simulated microgravity. Shear forces have been found to induce apoptosis in lung cancer cells via the Smad 1/5 pathway, inducing caspase 9 [25]. On the other hand, we observed an increase in apoptosis in lung cancer cells under reduced external forces together with an upregulation of apoptosis-related genes, such as *TP53*, *PTEN*, and RB1. We postulate that a cellular “gravity sensor” may exist, transforming a reduction in external force into apoptosis signals. Future identification of this “sensor” may provide options to selectively trigger tumor apoptosis in a therapeutic manner. Several pathways may be involved in this gravity sensing, but it is presumed that the cytoskeleton plays a crucial role, wherein the PTEN- PI-3-kinase pathway could be a key effector [26]. With its role in cytoskeleton formation and arrangement, the CDKN2A-p14 pathway is another possible candidate to mediate the effects of microgravity [27]. Further experiments that may include methods such as gene silencing are required to further elucidate this question. 

The terminal uracil-nicked end labeling (TUNEL) assay revealed a significantly higher proportion of apoptotic cells than non-viable cells on Trypan blue staining. Other authors have made similar discoveries. Gain et al. attributed this phenomenon to the higher sensitivity of the TUNEL assay, with cells taking up stain before changes could be detected by Trypan blue. [28]. As a result of the measurement time points chosen, it is conceivable that several cells entered apoptosis without compromising viability. Future viability experiments including later time points are required to answer this question. Pisanu et al. found an increased apoptosis rate under simulated microgravity in a non-small cell lung cancer cell line known to be rich in stem cells [20]. Exact mechanisms and underlying pathways have to be investigated in future studies. We noted a few significant visible cytoskeletal changes. First, actin filaments demonstrated rearrangement from a longitudinal pattern under 1-g-conditions in control cells to a spherical pattern in simulated microgravity. This is a documented phenomenon seen in other cell types [11,29]. Second, actin orientation in adherent cells under simulated microgravity was halfway between longitudinal and spherical, suggesting a transitional state. It is feasible that simulated microgravity first disrupts cell adherence, and continued exposure then induces a new 3D cell rearrangement into spheroids. Taken together, these findings lend credence to the postulated “gravity-sensing” function of the cytoskeleton [30]. It is known that filaments of polymerized actin are enhanced at focal adhesion sites, mediating intercellular contact [31], and may not only mediate the response of an individual cell to gravity but also facilitate the propagation of signals between cells at cell contact sites and may thus play a coordinating role in this process. The exact mechanisms behind this remain to be discovered. Further, actin filaments partake in the process of cell division, and disruption interferes with the correct orientation of the mitosis spindle [32]. This may explain a reduction in cell proliferation under simulated microgravity [33,34].

Selective modification of actin alignment in malignant cells by other molecular methods, such as RNA-interference or epigenetic modification, may pave the way towards new options in cancer therapy. This study has some limitations. Spheroids were collected by sedimentation, and efforts were taken to minimize the duration of the sedimentation period to minimize the effect of gravity. This reduced the number of sedimented spheroids and allowed determination of their actin structure qualitatively but was insufficient to quantify the ratio of longitudinally- to spherically-aligned cytoskeletons. This will be the subject of future investigative efforts. 

Some of the alterations in gene expression that were detected are indicative of an alteration in cell adherence mechanisms. PTEN upregulation has been shown to attenuate cell adhesion in osteosarcoma cells [35]. On the other hand, SOX2 overexpression suggests enhanced cell adhesion in human dental pulp stem cells [36]. The question of whether microgravity induces a more malignant or a more benign phenotype in tumor cells is still a subject of debate. 

Some investigators found a shift towards a more benign phenotype. Chang et al. reported decreased proliferation and decreased expression of MMP2 in lung cancer cells under simulated microgravity, suggesting a reduction in metastatic potential [21]. 

We found an upregulation of tumor suppressor genes in our experiments. *TP53*, *PTEN*, *RB1*, and *CDKN2A* have been identified as tumor suppressor genes in lung cancer [37]. The TP53 gene product is known to act as a tumor suppressor by binding to damaged DNA, arresting cell cycle to allow time for repair and to induce apoptosis in case of failed repair [38]. 

PTEN plays a role in cell adherence and is an important negative regulator of the cell-survival signaling pathway initiated by phosphatidylinositol3-kinase (PI3K). The PI3K pathway plays an important role in cell proliferation and cell survival, particularly in cells responsive to growth factors. [39]. *PTEN* upregulation under simulated microgravity, especially in the adherent cells, may indicate reduced tumor proliferation and survival. Several interactions exist between the PI3K-PTEN and the TP53 pathway. We observed an upregulation of *TP53* gene expression in the adherent cells and TP53 protein production in the spheroids, suggesting synergism between the two pathways. Additionally, TP53 increases the recycling of the adhesion molecule integrin α5β1 and therefore plays a role in adherence/invasion and metastasis [40]. Cyclin-dependent kinase CDKN2A inactivates cyclin dependent kinases 4 and 6 (CDK4 and CDK6), which in turn decrease phosphorylation of the pRb [41]. Unphosphorylated pRb blocks transcriptional activation of E2F and thereby attenuates cell proliferation [42]. We observed upregulation of both *CDKN2A* /P14 and *RB1*/RB1 tumor suppressors both at the gene and at the protein level under simulated microgravity. These changes may suggest transformation towards a less malignant phenotype under simulated microgravity. 

PI3K/AKT/mTOR is a pivotal signal transduction pathway involved in regulation of cell proliferation, adhesion, differentiation, and motility [39,43]. We found that the expression of oncogenes *AKT3*, NFE2L2, and *PIK3CA* as a catalytic subunit of phosphoinositide 3-kinase (PI3K) remained unchanged under simulated microgravity. The reasons for this are as yet unclear. It is possible that assessment at earlier or later timepoints might yield different results, as it is known that changes in gene expression under real and simulated microgravity can occur very rapidly, within minutes to hours [29,44,45]. It is thus possible that assessment at the 72 h timepoint may have been too late to detect these changes. However, we also observed an upregulation of the oncogene *SOX2. SOX2* and its downstream target *PIK3CA* are both located on the genomic region 3q and are known to play a crucial role in the initiation and the progression of squamous cell lung cancer. Its gene function seems to support squamous identity of the tumor cells and may therefore act as a “lineage survival oncogene” rather than a gene of ongoing dedifferentiation [46]. We observed increased gene upregulation and protein production of SOX2 under simulated microgravity, particularly in the adherent cells. This finding supports the theory of a more “in vivo-like” phenotype expression under 3D culture conditions. *PIK3CA,* as a downstream target of *SOX2,* was not upregulated in our experiments. As it is known that SOX2 is activated particularly in early stages of non-squamous lung cancer [47], activation of its downstream targets could still be forthcoming at our observation time point of 72 h. For some of our target genes and proteins, we found differences in gene and protein up- or down-regulation, respectively, namely for *TP53*/TP53, *PTEN*/PTEN, and *SOX2*/SOX2. These findings could be explained by the existence of various post-translational modifications, differing half-lives of proteins, rapid degradation of mRNA, or delayed protein synthesis [48]. Other authors have reported a general decrease in protein synthesis under microgravity for unknown reasons [49]. It is notable that we found changes in gene expression and protein production in the adherent cells under simulated microgravity that differed from those in the spheroids. It is possible that the adherent cell cohort may not just be an intermediate state or precursor of spheroids but may constitute a distinct entity with specific characteristics. Future long-term experiments are necessary to determine if these cells will react to simulated microgravity with delayed detachment (or loss of adherence) or even no detachment. As these long-term adherent cells may represent a less invasive phenotype, identifying the characteristics of this subgroup may provide further understanding into the mechanisms contributing to metastasis and invasion. Cell adherence and apoptosis are two particular features that may have a significant relevance with regards to designing new targeted therapies. Respective information may facilitate the future development of new therapeutic agents. With regards to future space travel, our results do not support that microgravity itself induces lung cancer in astronauts. It is difficult to directly extrapolate in vitro cell culture results to a whole organism, and many additional factors, including space radiation and changes in the immune system in the absence of gravity, neither of which could be explored in our study, may contribute to the risk of carcinogenesis [50,51]. Experiments in space involving animal models may bridge the gap between culture dish and whole organism and may further deepen our understanding of the effects of space on lung cancer growth. 

## 4. Materials and Methods

### 4.1. Cell Culture

Human lung cancer cells (squamous, non small-cell, cell line CRL-5889) were obtained from ATCC^©^ (Wesel, Germany). Cells were first expanded under 2D conditions in T75 flasks (Sarstedt, Newton, MA, USA). Ham’s F12-media (Gibco, Berlin, Germany) supplemented with 5% fetal calf serum (FCS) (Biochrom AG, Berlin, Germany) and 1% penicillin/streptomycin (Biochrom, Berlin, Germany) was used. The medium was changed three times a week. For the experiments, 1 × 10^6^ cells were counted by a hemocytometer and added to T75 flasks in the random positioning machine. An equal number of cells were kept under 1-g conditions as controls. For cytoskeleton staining, cells were seeded with a density of 1 × 10^5^ per cm^2^ into slide flasks (Thermo Scientific, Darmstadt, Germany). 

### 4.2. Cell Harvest for RNA and Protein Isolation

Attached cells from the control group and adherent cells from the experimental groups were detached from the culture flask surface by incubation with 0.05% trypsin (Thermo Scientific, Berlin, Germany) for 5 min. The enzymatic reaction was subsequently stopped with an equal amount of medium, and the remaining attached cells were loosened with a cell scraper (Thermo Scientific, Berlin, Germany). The suspension was then centrifuged at 2000 rpm for 5 min to obtain a cell pellet. Spheroids were harvested by pipetting off the liquid medium from the experimental group flasks and centrifuging the cells down at 2000 rpm for 5 min for RNA isolation. For actin staining, spheroids were collected by sedimentation. The sedimentation period was kept as short as possible to minimize the influence of gravity.

### 4.3. Random Positioning Machine

The RPM (developed by University of Applied Sciences, Northwestern Switzerland) was placed in a commercially available incubator at 37 °C and 5% CO_2_. The device was operated in a random walk modus using an angular velocity of 60°/s [52]. T75 flasks were attached to the center of the operating platform, and samples were rotated for the selected time period (72 h). Static, non-rotated controls were exposed to the same environmental conditions in proximity of the device. Care was taken to keep flasks free of air bubbles to avoid shear forces therefrom. The RPM machine was rebooted once every 24 h to ensure proper operation. Efforts were taken to minimize the duration of interruption. Experiments were performed 6 times independently, and results were reported as means and standard deviation.

### 4.4. Phase Contrast Microscopy

Phase contrast microscopy was performed for visual observation of cell viability and morphology and for the detection of potential spheroids. A Leica microscope (Leica Microsystems GmbH, Wetzlar, Germany) was used. Pictures were taken with a Canon EOS 60D (Canon GmbH, Krefeld, Germany). 

### 4.5. Trypan Blue Staining

Trypan blue stock solution was obtained from Thermo Fisher, Germany. Aliquots of the control and the experimental groups were taken at a volume of 0.5 mL and mixed with an equal amount of Trypan blue stock solution. The mixture was incubated for two minutes at room temperature and then loaded onto a hematocytometer in a volume of 10 μL. The cells were counted, and the ratio of viable to non-viable cells was calculated. 

### 4.6. Cytoskeleton Staining

Cells exposed for 72 h to simulated microgravity in the RPM in slideflasks were investigated. F-actin was visualized by means of phalloidin staining (PromoKine, High Point, NC, USA). Both adherent cells and spheroids were fixed with 4% paraformaldehyde for 10 min and permeabilized with 1% Triton-X for 5 min. Free floating spheroids were allowed to settle to the bottom of the slideflask for 10 min before fixation. Nonspecific binding was blocked by incubation with 1% bovine serum albumine (BSA). Staining was performed by incubation of the slides with 6.6 μM solution of a phalloidin/Alexa Fluor 488 conjugate for 30 min at room temperature followed by thorough washing with phosphate buffered saline (PBS) solution. Nuclei were counterstained with 4′,6-diamidine-2-phenylindol (DAPI) (Thermo Fisher Scientific, Berlin, Germany) at a concentration of 0.1 μg/mL for 1 min. The samples were mounted with Aqua Poly/Mount coverslipping medium (Polysciences, Eppelheim, Germany).

### 4.7. Fluoresence and Confocal Microscopy

Slides were first observed with an Axio Imager 2 fluorescence microscope (Carl Zeiss AG, Jena, Germany). Confocal microscopy of the slides stained for F-actin was performed using a Leica TCS SPE inverted confocal laser scanning microscope (Leica Camera AG, Wetzlar, Germany). Excitation and emission wavelengths were 485 nm and 560 nm, respectively. 

### 4.8. TUNEL Assay

The TACS 2 TdT-Fluor in Situ Apoptosis Detection Kit was obtained from Trevigen Systems, USA. After culturing the cells in slide flasks as described above, TUNEL assay was performed according to the manufacturer’s protocol. Both adherent cells and spheroids were fixed with 4% paraformaldehyde for 10 min. Samples were incubated with 50 μL of Proteinase K solution for 15 min. After washing with deionized water, slides were immersed with 1x TdT Labeling Buffer for 5 min. Thereafter, slides were incubated with 50 μL of the Labeling Reaction Mix in a moisture chamber. A positive control was provided by incubating a further slide with TACS Nuclease. After addition of Stop Buffer and washing with PBS, 50 μL of Strep-Fluor Solution was added for 20 min. Solution was washed off with PBS. Nuclei were counterstained with 1 μL of DAPI (5mg/mL) for cell counting (images not shown). Slides were closed with coverslips and fluorescence mounting media. Slides were observed under a fluorescence microscope at 495 nm wave length. Cells were counted on 10 fields of view at 100× magnification for each group, and the ratio of apoptotic to non-apoptotic cells was calculated. TUNEL assays were performed after 24 h and after 72 h exposure to simulated microgravity.

### 4.9. RNA and Protein Isolation and Quantitative Real-Time PCR

#### 4.9.1. RNA Isolation

An aliquot of cells was frozen in liquid nitrogen for subsequent lysis and protein isolation as described below. RNA isolation and quantitative real-time polymerase chain reaction (qRT-PCR) were performed according to routine protocols described in the manufacturer’s manual. RNA was isolated using the RNeasy^©^ Kit (Qiagen, Hilden, Germany) following the manual’s instructions. Cells were detached from the culture plates with 0.05% trypsin (Sigma-Aldrich, Hamburg, Germany). The cell suspension was centrifuged in an RNase-free tube for 5 min at 300 g. The pellet was lysed by adding 350 μL of the lysis buffer (RLT, Qiagen, Hilden, Germany). Cell-inherent RNases were inactivated by adding 1% β-mercaptoethanol. The lysate was homogenized by vortexing for 1 min. It was then stabilized by adding 1 volume of 70% ethanol. The solution was then added to an RNeasy Spin Column and centrifuged in a microcentrifuge at 10,000 rpm for 15 s. The flow-through was discarded. Subsequently, 700 μL of the washing buffer RW1 was added to the spin column, and the column was centrifuged again at 10,000 rpm for 15 s, and the flow-through was discarded. Five hundred microliters of the washing buffer RPE were added to the spin column and centrifuged at 10,000 rpm for 15 s. This step was repeated after discarding the flow-through. The spin column was then placed into an RNase-free collection tube, and the RNA was eluted with 30 μL of RNase-free water. The RNA was quantified photometrically with the SpectraMax M2 device (Molecular Devices, Sunnyvale, CA, USA). The RNA amount was determined by measuring the absorbance at 260 nm. 

#### 4.9.2. Reverse Transcription

Reverse transcription was performed using the First Strand cDNA Synthesis Kit (Thermo Scientific, Waltham, MA, USA) following manufacturer’s instructions. For each sample, 1 μg total RNA, random hexamer primers, and nuclease-free water were added to a total volume of 11 μL. To this mix, reaction buffer, RNase inhibitor, oligonucleotides, and reverse transcriptase were added to a total volume of 20 μL. All steps were carried out on ice. The solution was incubated for 5 min at 25 °C followed by 42 °C for 60 min. The reaction was terminated by incubation at 70 °C for 5 min. The cDNA solution was stored at −20 °C for less than one week before proceeding with the experiments. 

#### 4.9.3. Real-Time PCR

Quantitative real-time polymerase chain reaction was utilized to determine the expression levels of target genes, shown in Table 1, using the SYBR^®^ Green PCR Master Mix (Applied Biosystems, Darmstadt, Germany) and the 7500 Real-Time PCR System (Applied Biosystems). Then, 22.5 μL master mix, 0.15 μL of forward and reverse primer (each at a concentration of 100 μM), and 3 μL of cDNA and RNase free water (dependent on the input-amount of RNA) were added. The following cycling steps were performed after activation of uracil-DNA gylcosylase (50 °C for 2 min) and DNA polymerase (95 °C for 2 min): 95 °C for 15 s and 60 °C for 1 min (40 cycles). Specific amplification was confirmed by dissociation curves. cDNA-selective primers were identified from Harvard primer database (https://pga.mgh.harvard.edu/primerbank), except for *EEF1A1,* which was identified from the literature [53], and the primers were synthesized by TIB Molbiol (Berlin, Germany). All samples were measured in triplicate and normalized to the housekeeping gene *EEF1A1*. The comparative C_T_ (ΔΔC_T_) method was used for relative quantification of transcription levels with the control group set as 100%. Primer sequences were as indicated in Table 1.

### 4.10. Western Blotting

Gel electrophoresis, transblotting, and densitometry were performed according to standard protocols described in detail elsewhere [54]. Cell lysate was produced by adding lysis buffer to cell pellets; 10 μL of cell lysate containing 1.2 μg/μL protein were loaded on SDS-PAGE. The cell homogenate protein was incubated for 10 min with SDS-gel loading buffer (1 M Tris base, pH 6.8; 1% glycerol; 10% SDS; 0.1% bromophenol blue; freshly added with 0.05% β-mercaptoethanol and 1% protease inhibitors; Complete, Roche). Samples were denatured at 95 °C for 5 min. Afterwards, probes were loaded together with the pre-stained protein ladder (Protein C Western Standard, BioRad Laboratories, Munich, Germany) onto a 10% SDS-polyacrylamide gel after electrophoresis and semi-dry blotting onto 0.45 μm nitrocellulose membranes (BioRad Laboratories, Munich, Germany). The following primary antibodies (all obtained from Santa Cruz Biotechnology, Paso Robles, CA, USA) were used in the blocking reagent in 1:400 dilution: mouse anti-tp53, anti-p14, anti-RB1, anti-PTEN, and anti-SOX2. Horse-radish peroxidase coated M-IgGκ anti-mouse was used as a secondary antibody at a dilution of 1:1000. Normalization was performed using total protein analysis as described elsewhere [55]. Afterwards, the blots were blocked with milk powder and washed. Blots were analyzed on a ChemiDoc XRS+ System (BioRad Laboratories, Munich, Germany).

## 5. Conclusions

Simulated microgravity induces alteration in cell adherence, increases apoptosis rates, and leads to upregulation of tumor suppressor genes in human lung cancer cells. These findings may be important in the field of medical oncology. A deeper understanding of the mechanisms at play may contribute to the development of novel therapeutic strategies in our crusade against cancer. 

## Figures and Tables

**Table 1:** All primers where indentified from the *Harvard Primer Database*, except *EEF1A1.*

Primer	Gene
Fwd 5′CAGCACATGACGGAGGTTGT3′ Rev 5′TCATCCAAATACTCCACACGC3′	TP53
Fwd 5′GATCCAGGTGGGTAGAAGGTC3′ Rev 5′CCCCTGCAAACTTCGTCCT3′	CDKN2A
Fwd 5′CTCTCGTCAGGCTTGAGTTTG3′ Rev 5′GACATCTCATCTAGGTCAACTGC3′	*RB1*
Fwd 5′TGGATTCGACTTAGACTTGACCT3′ Rev 5′GGTGGGTTATGGTCTTCAAAAGG3′	*PTEN*
Fwd 5′TGGACAGTTACGCGCACAT3′ Rev 5′CGAGTAGGACATGCTGTAGGT3′	*SOX2*
Fwd 5′TGTGGATTTACCTTATCCCCTCA3′ Rev 5′GTTTGGCTTTGGTCGTTCTGT3′	*AKT3*
Fwd 5′CCACGACCATCATCAGGTGAA3′ Rev 5′CCTCACGGAGGCATTCTAAAGT3′	*PIK3CA*
Fwd 5′TCAGCGACGGAAAGAGTATGA3′ Rev 5′CCACTGGTTTCTGACTGGATGT3′	*NFE2L2*
Fwd 5′CATCAAAGCAGTGGACAAGAAG3′ Rev 5′GGGTGGCAGGTATTAGGGATAA3′	*EEF1A1* [53]

## Abbreviations

2D	two-dimensional
3D	three-dimensional
AKT3	AKT serine/threonine kinase 3
CDKN2A	cyclin dependent kinase inhibitor 2A
cDNA	complementary desoxyribonucleic acid
DAPI	4′,6-diamidine-2-phenylindol
DNA	desoxyribonucleic acid
EEF1A1	elongation factor 1-alpha 1
kDa	kDa kilodalton
MW	MW molecular weight
*NFE2L2*	NFE2L2 nuclear factor (erythroid-derived 2)-like 2
PBS	phosphate-buffered saline
PTEN	phosphatase and tensin homolog
PIK3CA	phosphatidylinositol-4,5-bisphosphate 3-kinase catalytic subunit alpha
qRT-PCR	quantitative real time polymerase chain reaction
RB1	retinoblastom protein 1
RNA	ribonucleic acid
RPM	random positioning machine
rpm	rounds per minute
SOX2	sex determining region Y (SRY)-box
TUNEL	terminal uracil-nicked end labeling
